# Optimizing CAR T cell therapy in pancreatic cancer through clinically actionable combinations

**DOI:** 10.1016/j.omton.2026.201231

**Published:** 2026-05-28

**Authors:** Giulia Escobar, Marcela V. Maus

**Affiliations:** 1 Krantz Family Center for Cancer Research, Massachusetts General Hospital, Charlestown, MA, USA; 2 Cellular Immunotherapy Program, Massachusetts General Hospital, Charlestown, MA, USA; 3 Harvard Medical School, Boston, MA, USA

## Main text

Pancreatic ductal adenocarcinoma (PDAC) remains one of the most refractory malignancies to immunotherapy, owing to physical and immunologic barriers that collectively limit the efficacy of T cell-based approaches, including chimeric antigen receptor (CAR) T cell therapies. In PDAC, a dense desmoplastic stroma restricts T cell infiltration, while immunosuppressive factors derived from tumor cells, stromal components, and regulatory immune populations (i.e., regulatory T cells and suppressive myeloid cells) actively impair T cell function.^1^ As a result, therapeutic efficacy depends not only on tumor recognition but also on the ability of engineered T cells to infiltrate tumors and sustain functional activity within a highly suppressive tumor microenvironment (TME).

**Figure 1:**
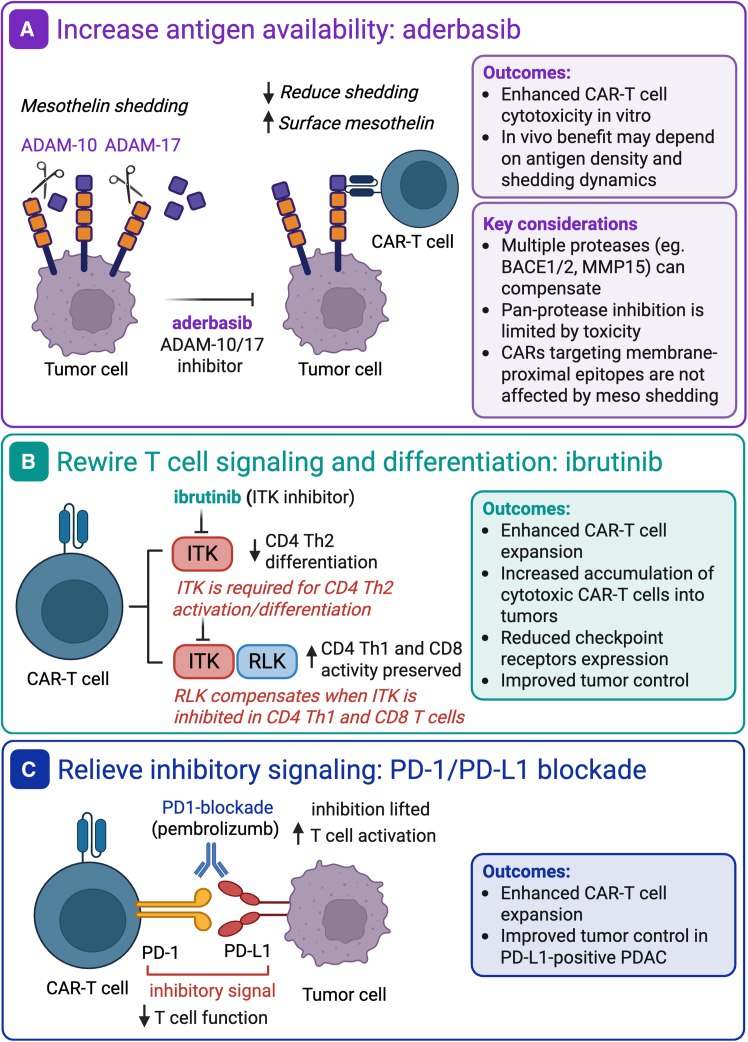
Combination approaches to enhance CAR-T cell efficacy in PDAC (A) Mesothelin is shed by ADAM-10 and ADAM-17. Truncated mesothelin is not recognized by SS1-mesothelin-targeting CAR T cells. Treatment with the ADAM-10/17 inhibitor aderbasib reduces mesothelin shedding and increases surface antigen density. This results in enhanced tumor killing by CAR T cells *in vitro* but not *in vivo*, suggesting context-dependent benefit and redundancy among proteolytic pathways. (B) Ibrutinib inhibits ITK, a kinase required for Th2 differentiation. In Th1 and CD8 T cells, compensatory RLK signaling preserves activation thereby promoting a Th1-skewed state associated with enhanced CAR T cell expansion and function. (C) PD-1 blockade restores CAR T-cell function and enhances antitumor efficacy in PDL1-positive PDAC tumors. Created in Biorender. Escobar, G. (2026). https://Biorender.com/97x5a9m.

To address this challenge in a recent study published in *Clinical Cancer Research*, we explore combinatorial strategies that simultaneously address tumor-intrinsic and T cell-intrinsic limitations to enhance CAR T cell efficacy against PDAC.^2^ Using mesothelin-targeting CAR T cells in preclinical models of PDAC, we systematically evaluated drug combinations designed to modulate antigen availability, enhance T cell functional state, and relieve inhibitory signaling in the TME (Figure 1).

One strategy to improve tumor targeting is to increase target antigen density (Figure 1A). Mesothelin, a cell surface protein overexpressed in PDAC, undergoes proteolytic shedding mediated by tumor-derived proteases, including ADAM-10 and ADAM-17, reducing mesothelin availability for CAR engagement while also generating soluble mesothelin ectodomains that may act as decoys.^3^^,^^4^ Pharmacologic inhibition of ADAM-10 and ADAM-17 proteases with aderbasib resulted in a dose-dependent increase in surface mesothelin expression and a reduction in soluble antigen levels *in vitro*. Consistent with this mechanism, CAR T cell cytotoxicity was enhanced in co-culture assays. However, these effects did not translate into improved tumor control *in vivo*. In our model, mesothelin expression was already high *in vivo*, suggesting that limiting antigen shedding did not confer additional therapeutic benefit. Rather than indicating a general limitation of antigen modulation strategies, these findings suggest that its benefit may be restricted to settings in which antigen density is suboptimal or highly dynamically regulated. Nonetheless, the use of protease-specific inhibitors is also limited by the redundancy among proteolytic pathways, which can lead to compensatory activity, while the use of pan-protease inhibitors is constrained by systemic toxicity. Therefore, alternative strategies, such as designing CARs that recognize membrane-proximal epitopes of mesothelin not susceptible to cleavage,^5^ may offer a more effective solution than pharmacologic inhibition.

In contrast, interventions that reshape T cell functional state resulted in more effective enhancement of therapeutic efficacy. We found that modulation of T cell-intrinsic signaling pathways with the small molecule drug, ibrutinib, resulted in an additive effect (Figure 1B). Ibrutinib, an inhibitor of Bruton tyrosine kinase (BTK) and IL2-inducible T cell kinase (ITK), has been extensively employed in B cell malignancies, where it exerts a direct anti-tumor effect through the inhibition of the BTK kinase downstream the B cell receptor. Besides its direct effect on malignant BTK-positive B cells, ibrutinib was also shown to affect T cell signaling and functionality through ITK inhibition, promoting a shift toward Th1 polarization.^6^^,^^7^ In BTK-negative PDAC tumors, we showed that combination of CAR T cell therapy with ibrutinib treatment translated into enhanced expansion and accumulation of cytotoxic CAR T cells within tumors and was associated with a more favorable functional state, characterized by increased persistence and reduced upregulation of exhaustion-associated markers. Mechanistically, ITK inhibition selectively impairs Th2 CD4 T cell differentiation while preserving Th1 CD4 and cytotoxic CD8 T cell responses, effectively re-balancing the immune program toward a phenotype more compatible with sustained antitumor activity. Notably, the benefit was most evident under conditions of limiting CAR T cell dose, a scenario that may more closely reflect the clinical setting, where autologous T cells are often functionally compromised and numerically constrained in patients with pancreatic tumors. These observations indicate that maintaining the balance between T cell activation and overstimulation is critical for durable CAR T cell responses, and that by limiting activation-induced cell death while supporting expansion, ibrutinib may help preserve functional effector cells capable of sustaining antitumor activity. These findings extend prior observations in BTK-positive hematologic malignancies^6^^,^^7^ into a BTK-negative solid tumor setting and highlight the potential of repurposing clinically approved agents to enhance CAR T cell fitness.

A third axis of intervention involves the blockade of inhibitory signaling within the TME (Figure 1C). Programmed cell death ligand 1 (PD-L1) is upregulated on a third of PDAC tumors and is associated with reduced overall survival, yet checkpoint blockade alone has shown limited clinical benefit, largely due to the paucity of endogenous tumor-reactive T cells in PDAC tumors.^8^ However, rather than relying on the reinvigoration of endogenous immune responses, PD-1 blockade in the context of adoptive cell therapy may act directly on transferred CAR T cells, restoring effector function upon antigen encounter on PD-L1-positive tumor cells. In a patient-derived xenograft model that expresses high levels of PDL1, we found that PD-1 blockade synergistically enhanced CAR T cell-mediated tumor control and was associated with improved CAR T cell expansion, effectively leveraging inhibitory pathways in an immunologically “cold” tumor.

In parallel, our study further highlights the importance of delivery strategies in shaping therapeutic outcomes. While CAR T cells are typically administered systemically, locoregional delivery may provide a means to overcome physical barriers and increase effective tumor exposure, as recently shown in clinical trials for solid tumors.^9^ Using a model of peritoneal disease, we found that intraperitoneal administration of CAR T cells resulted in superior tumor control compared with intravenous delivery and was accompanied by enhanced expansion and reduced expression of inhibitory markers. These findings suggest that optimizing the spatial distribution of CAR T cells could further amplify the effects of functional reprogramming.

From a translational perspective, these results provide a framework for prioritizing clinically actionable combinations. Looking forward, the next phase of development will likely involve integrating these pharmacologic strategies with advances in cellular engineering. In this regard, combination of these drugs with CAR T cells engineered to co-target mesothelin-positive tumor cells through the CAR- and FAP-positive cancer-associated fibroblasts through a T cell-secreted FAP-CD3 T cell engager is in clinical development.^10^ In the future, genetic approaches that enhance CAR T cell metabolic fitness, promote durable functional states, enable multi-antigen targeting, and incorporate armoring strategies to counteract the immunosuppressive TME could be combined with systemic therapies to further reinforce CAR T cell function. Emerging tools such as CRISPR-based screens and high-dimensional profiling are already enabling systematic mapping of the regulatory networks that govern T cell behavior within tumors, providing a foundation for the more precise design of next-generation cellular therapies.^9^

## Acknowledgments

This work was supported by UG3UCA283619, awarded to M.V.M. and the 10.13039/100005564Gilead Sciences Research Scholar Program awarded to G.E.

## Declaration of interests

M.V.M. is an inventor on patents related to adoptive cell therapies, held by Massachusetts General Hospital (some licensed to Promab, Luminary, and Altido Therapeutics) and University of Pennsylvania (some licensed to Novartis). He receives grant/research support from BMS, Kite Pharma, Miltenyi, Sobi. M.V.M. also holds equity in Altido Therapeutics and Umoja BioPharma and serves as the Board of Directors for Umoja BioPharma. M.V.M. is a compensated Consultant for A2Bio (SAB), Alexion, Astellas, AstraZeneca, BMS, Cabaletta Bio (SAB), Chugai, Healio, KSQ, Lumicks, Triacyte. M.V.M.’s interests (particularly in A2Bio and Altido Therapeutics) were reviewed and are managed by MGH and Mass General Brigham in accordance with their conflict of interest policies.

## References

[1] Halbrook C.J., Lyssiotis C.A., Pasca di Magliano M., Maitra A. (2023). Pancreatic cancer: Advances and challenges. Cell.

[2] Armstrong A., van der Plancke G., Nishiguchi S., Salas-Benito D., Bouffard A.A., Goncalves S., Merce A.T., Kelly C., Birocchi F., Park S. (2026). Ibrutinib and PD-1 Blockade Potentiate Mesothelin-Targeting CAR T-cell Therapy in Preclinical Models of Pancreatic Cancer. Clin. Cancer Res..

[3] Liu X., Chan A., Tai C.-H., Andresson T., Pastan I. (2020). Multiple proteases are involved in mesothelin shedding by cancer cells. Commun. Biol..

[4] Zhang J., Qiu S., Zhang Y., Merino M., Fetsch P., Avital I., Filie A., Pastan I., Hassan R. (2012). Loss of mesothelin expression by mesothelioma cells grown in vitro determines sensitivity to anti-mesothelin immunotoxin SS1P. Anticancer Res..

[5] Liu X., Onda M., Watson N., Hassan R., Ho M., Bera T.K., Wei J., Chakraborty A., Beers R., Zhou Q. (2022). Highly active CAR T cells that bind to a juxtamembrane region of mesothelin and are not blocked by shed mesothelin. Proc. Natl. Acad. Sci. USA.

[6] Fraietta J.A., Beckwith K.A., Patel P.R., Ruella M., Zheng Z., Barrett D.M., Lacey S.F., Melenhorst J.J., McGettigan S.E., Cook D.R. (2016). Ibrutinib enhances chimeric antigen receptor T-cell engraftment and efficacy in leukemia. Blood.

[7] Dubovsky J.A., Beckwith K.A., Natarajan G., Woyach J.A., Jaglowski S., Zhong Y., Hessler J.D., Liu T.-M., Chang B.Y., Larkin K.M. (2013). Ibrutinib is an irreversible molecular inhibitor of ITK driving a Th1-selective pressure in T lymphocytes. Blood.

[8] Hu Y., Chen W., Yan Z., Ma J., Zhu F., Huo J. (2019). Prognostic value of PD-L1 expression in patients with pancreatic cancer: A PRISMA-compliant meta-analysis. Medicine.

[9] Escobar G., Berger T., Maus M. (2025). CAR-T cells in solid tumors: Challenges and breakthroughs. Cell Rep. Med..

[10] Wehrli M., Guinn S., Birocchi F., Kuo A., Sun Y., Larson R.C., Almazan A.J., Scarfò I., Bouffard A.A., Bailey S.R. (2024). Mesothelin CAR T-cells secreting anti-FAP/anti-CD3 molecules efficiently target pancreatic adenocarcinoma and its stroma. Clin. Cancer Res..

